# Supplementary material for: Single Cell Transcriptomic and Chromatin Profiles Suggest Layer Vb Is the Only Layer With Shared Excitatory Cell Types in the Medial and Lateral Entorhinal Cortex
Source: Front Neural Circuits. 2022 Jan 26;15:806154. doi: 10.3389/fncir.2021.806154 (PMC8826650; doi:10.3389/fncir.2021.806154)
Supplement: Supplementary file 14 [file Data_Sheet_1.ZIP › MEC_Sup_vs_LEC_Sup_Motifs/knownResults.html]

MEC\_Sup\_vs\_LEC\_Sup\_Motifs/ - Homer Known Motif Enrichment Results


# Homer Known Motif Enrichment Results (MEC\_Sup\_vs\_LEC\_Sup\_Motifs/)

Homer *de novo* Motif Results  
Gene Ontology Enrichment Results  
Known Motif Enrichment Results (txt file)  
Total Target Sequences = 21074, Total Background Sequences = 12206

|  |  |  |  |  |  |  |  |  |  |  |  |
| --- | --- | --- | --- | --- | --- | --- | --- | --- | --- | --- | --- |
| Rank | Motif | Name | P-value | log P-pvalue | q-value (Benjamini) | # Target Sequences with Motif | % of Targets Sequences with Motif | # Background Sequences with Motif | % of Background Sequences with Motif | Motif File | SVG |
| 1 | A T G C T G C A A G T C C G T A A G T C A G T C A C G T A C T G A T G C G T C A | E2A(bHLH),near\_PU.1/Bcell-PU.1-ChIP-Seq(GSE21512)/Homer | 1e-2102 | -4.842e+03 | 0.0000 | 13652.0 | 64.78% | 3871.7 | 31.72% | motif file (matrix) | svg |
| 2 | T G C A G T A C C G T A A T C G A C T G A C G T C T A G C G A T T C G A A G T C | ZEB1(Zf)/PDAC-ZEB1-ChIP-Seq(GSE64557)/Homer | 1e-1603 | -3.691e+03 | 0.0000 | 14000.0 | 66.43% | 4541.1 | 37.20% | motif file (matrix) | svg |
| 3 | A G C T T C G A G T A C T C G A A T G C A T G C G C A T A T C G A G T C A G C T | Snail1(Zf)/LS174T-SNAIL1.HA-ChIP-Seq(GSE127183)/Homer | 1e-1570 | -3.617e+03 | 0.0000 | 10167.0 | 48.24% | 2647.7 | 21.69% | motif file (matrix) | svg |
| 4 | T A C G C T A G T A C G A G T C C G T A A G T C A G T C A C G T A C T G A G T C G A T C T A G C | Slug(Zf)/Mesoderm-Snai2-ChIP-Seq(GSE61475)/Homer | 1e-1372 | -3.161e+03 | 0.0000 | 7555.0 | 35.85% | 1704.5 | 13.96% | motif file (matrix) | svg |
| 5 | C G T A T G A C T C G A A G T C C G T A A T C G A T G C A C G T A C T G A G T C | E2A(bHLH)/proBcell-E2A-ChIP-Seq(GSE21978)/Homer | 1e-1204 | -2.774e+03 | 0.0000 | 13171.0 | 62.50% | 4535.2 | 37.15% | motif file (matrix) | svg |
| 6 | C T A G A C T G T G C A A G T C C G T A A C T G A C T G A C G T C T A G C G A T T A C G A G T C | ZEB2(Zf)/SNU398-ZEB2-ChIP-Seq(GSE103048)/Homer | 1e-1118 | -2.575e+03 | 0.0000 | 9051.0 | 42.95% | 2560.3 | 20.97% | motif file (matrix) | svg |
| 7 | T G A C A T G C C G T A A T C G A T G C C A G T C A T G A C T G A G T C G T A C | HEB(bHLH)/mES-Heb-ChIP-Seq(GSE53233)/Homer | 1e-1057 | -2.436e+03 | 0.0000 | 15148.0 | 71.88% | 5885.7 | 48.21% | motif file (matrix) | svg |
| 8 | T A C G T A C G C T A G T C A G A G T C C G T A A T C G A T G C A C G T A C T G A G T C G A C T | Ascl2(bHLH)/ESC-Ascl2-ChIP-Seq(GSE97712)/Homer | 1e-823 | -1.895e+03 | 0.0000 | 10695.0 | 50.75% | 3711.9 | 30.41% | motif file (matrix) | svg |
| 9 | T C G A A G T C C G T A A T C G T A G C A C G T A C T G A G C T A C G T A G T C | Ptf1a(bHLH)/Panc1-Ptf1a-ChIP-Seq(GSE47459)/Homer | 1e-747 | -1.721e+03 | 0.0000 | 17373.0 | 82.43% | 7830.1 | 64.14% | motif file (matrix) | svg |
| 10 | C T A G A G T C T A C G T A C G T G A C C G T A A C T G T A G C G C A T C A T G A T G C A G C T | Ascl1(bHLH)/NeuralTubes-Ascl1-ChIP-Seq(GSE55840)/Homer | 1e-361 | -8.321e+02 | 0.0000 | 12553.0 | 59.56% | 5564.8 | 45.59% | motif file (matrix) | svg |
| 11 | T C G A A G T C C G T A A T C G A T G C C G A T A C T G A G T C A G C T A C T G | Tcf12(bHLH)/GM12878-Tcf12-ChIP-Seq(GSE32465)/Homer | 1e-144 | -3.338e+02 | 0.0000 | 7382.0 | 35.03% | 3291.8 | 26.97% | motif file (matrix) | svg |
| 12 | T C G A T C G A A G T C C G T A C T A G T A G C A C G T A C T G | MyoG(bHLH)/C2C12-MyoG-ChIP-Seq(GSE36024)/Homer | 1e-121 | -2.796e+02 | 0.0000 | 7921.0 | 37.58% | 3664.7 | 30.02% | motif file (matrix) | svg |
| 13 | T G A C A G T C C G T A A C T G G T A C A C G T A C T G A C G T G A C T G A T C | Twist2(bHLH)/Myoblast-Twist2.Ty1-ChIP-Seq(GSE127998)/Homer | 1e-97 | -2.248e+02 | 0.0000 | 13186.0 | 62.57% | 6765.4 | 55.42% | motif file (matrix) | svg |
| 14 | T G A C G T A C C G T A A C T G T G A C C G A T A C T G A T C G A G C T T A C G T C G A T A G C G T A C C G T A A T C G T G A C G C A T A C T G A C T G A T G C | Twist(bHLH)/HMLE-TWIST1-ChIP-Seq(Chang\_et\_al)/Homer | 1e-75 | -1.746e+02 | 0.0000 | 1391.0 | 6.60% | 476.3 | 3.90% | motif file (matrix) | svg |
| 15 | T A C G T G C A A G T C C G T A A C G T T G A C A C G T A C T G A C T G G C A T | TCF4(bHLH)/SHSY5Y-TCF4-ChIP-Seq(GSE96915)/Homer | 1e-71 | -1.644e+02 | 0.0000 | 12068.0 | 57.26% | 6238.7 | 51.11% | motif file (matrix) | svg |
| 16 | C T A G T C G A T G A C A G T C C G T A A C T G G T A C A C G T A C T G A C T G | BHLHA15(bHLH)/NIH3T3-BHLHB8.HA-ChIP-Seq(GSE119782)/Homer | 1e-62 | -1.447e+02 | 0.0000 | 11472.0 | 54.43% | 5939.4 | 48.65% | motif file (matrix) | svg |
| 17 | A C T G A G T C G T C A C G T A A G T C C G T A C T A G C T A G G A C T C A T G | SCRT1(Zf)/HEK293-SCRT1.eGFP-ChIP-Seq(Encode)/Homer | 1e-60 | -1.389e+02 | 0.0000 | 2137.0 | 10.14% | 862.7 | 7.07% | motif file (matrix) | svg |
| 18 | A C T G T G A C G T A C C G T A A G T C T A C G A C G T A C T G G T C A A G T C | NPAS2(bHLH)/Liver-NPAS2-ChIP-Seq(GSE39860)/Homer | 1e-58 | -1.342e+02 | 0.0000 | 6452.0 | 30.61% | 3133.0 | 25.66% | motif file (matrix) | svg |
| 19 | C T G A T G A C T G A C C G T A A C G T T G A C A G C T C T A G A C G T G A C T | Olig2(bHLH)/Neuron-Olig2-ChIP-Seq(GSE30882)/Homer | 1e-58 | -1.341e+02 | 0.0000 | 13997.0 | 66.42% | 7451.4 | 61.04% | motif file (matrix) | svg |
| 20 | A C G T T C G A T C G A A G T C G T C A T A C G A T G C A C G T A C T G A G C T | Myf5(bHLH)/GM-Myf5-ChIP-Seq(GSE24852)/Homer | 1e-53 | -1.226e+02 | 0.0000 | 5822.0 | 27.63% | 2814.2 | 23.05% | motif file (matrix) | svg |
| 21 | T C A G T G A C G T A C C G T A A C G T T G A C A C G T T C A G A G C T G A C T | NeuroD1(bHLH)/Islet-NeuroD1-ChIP-Seq(GSE30298)/Homer | 1e-52 | -1.220e+02 | 0.0000 | 8126.0 | 38.56% | 4089.3 | 33.50% | motif file (matrix) | svg |
| 22 | G C A T C G T A C G A T A C T G A G T C G C T A C T G A C G T A C A G T A C T G C G T A T C A G | Oct6(POU,Homeobox)/NPC-Pou3f1-ChIP-Seq(GSE35496)/Homer | 1e-49 | -1.135e+02 | 0.0000 | 3801.0 | 18.04% | 1750.8 | 14.34% | motif file (matrix) | svg |
| 23 | T C G A T G A C G T A C C G T A C A G T T G A C A C G T A C T G A G C T A G C T | NeuroG2(bHLH)/Fibroblast-NeuroG2-ChIP-Seq(GSE75910)/Homer | 1e-48 | -1.106e+02 | 0.0000 | 12414.0 | 58.90% | 6579.9 | 53.90% | motif file (matrix) | svg |
| 24 | G A C T C G T A A C G T A C T G A G T C C G T A C T G A C G T A C A G T A C G T G T C A T C A G | Brn1(POU,Homeobox)/NPC-Brn1-ChIP-Seq(GSE35496)/Homer | 1e-47 | -1.105e+02 | 0.0000 | 2977.0 | 14.13% | 1326.5 | 10.87% | motif file (matrix) | svg |
| 25 | A C G T C T A G C G T A A G T C G T A C A C G T A C G T A C G T G T C A G T A C T G A C G A C T | Nur77(NR)/K562-NR4A1-ChIP-Seq(GSE31363)/Homer | 1e-46 | -1.072e+02 | 0.0000 | 1225.0 | 5.81% | 461.1 | 3.78% | motif file (matrix) | svg |
| 26 | T A C G A T C G T A G C G A T C A C T G A C G T A G T C A C G T C T A G A T C G | Smad4(MAD)/ESC-SMAD4-ChIP-Seq(GSE29422)/Homer | 1e-44 | -1.018e+02 | 0.0000 | 8768.0 | 41.60% | 4505.4 | 36.91% | motif file (matrix) | svg |
| 27 | T C A G A C G T T C G A T A G C A G T C C G T A A C T G G T A C A C G T A C T G A T C G A G T C | Atoh1(bHLH)/Cerebellum-Atoh1-ChIP-Seq(GSE22111)/Homer | 1e-42 | -9.757e+01 | 0.0000 | 9685.0 | 45.95% | 5040.0 | 41.29% | motif file (matrix) | svg |
| 28 | A G C T A C G T A C T G A T G C A G T C C G T A C T G A T A C G | NF1-halfsite(CTF)/LNCaP-NF1-ChIP-Seq(Unpublished)/Homer | 1e-41 | -9.668e+01 | 0.0000 | 9850.0 | 46.74% | 5136.9 | 42.08% | motif file (matrix) | svg |
| 29 | C G T A A C G T A G C T C G A T C T A G G T A C C G T A A G C T C G T A G C T A | Oct4(POU,Homeobox)/mES-Oct4-ChIP-Seq(GSE11431)/Homer | 1e-38 | -8.928e+01 | 0.0000 | 4088.0 | 19.40% | 1953.2 | 16.00% | motif file (matrix) | svg |
| 30 | A C T G G T C A A C G T G C A T C G A T T C A G G T A C G T C A A C G T C T G A | Oct11(POU,Homeobox)/NCIH1048-POU2F3-ChIP-seq(GSE115123)/Homer | 1e-38 | -8.899e+01 | 0.0000 | 2990.0 | 14.19% | 1372.3 | 11.24% | motif file (matrix) | svg |
| 31 | A T G C G A C T A C T G C A G T G A T C A C G T T A C G T A C G | Smad2(MAD)/ES-SMAD2-ChIP-Seq(GSE29422)/Homer | 1e-37 | -8.659e+01 | 0.0000 | 8432.0 | 40.01% | 4360.3 | 35.72% | motif file (matrix) | svg |
| 32 | A G C T G C A T A C T G A C G T A G T C A C G T C T A G T A C G | Smad3(MAD)/NPC-Smad3-ChIP-Seq(GSE36673)/Homer | 1e-36 | -8.485e+01 | 0.0000 | 13756.0 | 65.27% | 7448.3 | 61.01% | motif file (matrix) | svg |
| 33 | G T C A G C A T G C T A C A G T C T A G G A T C C G T A C T G A C G T A C G A T | Oct2(POU,Homeobox)/Bcell-Oct2-ChIP-Seq(GSE21512)/Homer | 1e-33 | -7.633e+01 | 0.0000 | 2565.0 | 12.17% | 1175.7 | 9.63% | motif file (matrix) | svg |
| 34 | G C A T C T A G G T A C A G T C C G A T A C T G C T A G C T A G G T A C G C T A | ZNF416(Zf)/HEK293-ZNF416.GFP-ChIP-Seq(GSE58341)/Homer | 1e-31 | -7.325e+01 | 0.0000 | 6934.0 | 32.90% | 3559.7 | 29.16% | motif file (matrix) | svg |
| 35 | C T G A C T A G A T C G A G C T A C T G G A C T A G T C C T G A | Tbx5(T-box)/HL1-Tbx5.biotin-ChIP-Seq(GSE21529)/Homer | 1e-31 | -7.224e+01 | 0.0000 | 15496.0 | 73.53% | 8528.3 | 69.86% | motif file (matrix) | svg |
| 36 | G A C T C T A G A T G C A G T C G T C A T A C G A T G C A T C G | HIC1(Zf)/Treg-ZBTB29-ChIP-Seq(GSE99889)/Homer | 1e-30 | -7.111e+01 | 0.0000 | 10269.0 | 48.73% | 5460.4 | 44.73% | motif file (matrix) | svg |
| 37 | G A T C C G T A G A C T C T A G G A T C C T G A G A C T C T G A G A C T C T A G G A T C C T G A G A C T C T G A G A C T | OCT:OCT(POU,Homeobox)/NPC-OCT6-ChIP-Seq(GSE43916)/Homer | 1e-29 | -6.815e+01 | 0.0000 | 1257.0 | 5.96% | 523.6 | 4.29% | motif file (matrix) | svg |
| 38 | C T G A T C A G G T A C G C T A A C T G T G A C G C A T C A T G | SCL(bHLH)/HPC7-Scl-ChIP-Seq(GSE13511)/Homer | 1e-28 | -6.644e+01 | 0.0000 | 18346.0 | 87.05% | 10291.8 | 84.31% | motif file (matrix) | svg |
| 39 | A T G C G A T C C G A T C T A G A C T G G C T A C G T A A G C T A C T G A G C T | TEAD2(TEA)/Py2T-Tead2-ChIP-Seq(GSE55709)/Homer | 1e-24 | -5.625e+01 | 0.0000 | 3211.0 | 15.24% | 1563.2 | 12.80% | motif file (matrix) | svg |
| 40 | G C T A A C G T C T A G G T A C G C T A G A C T C T G A G C A T C A T G G A T C | Pit1(Homeobox)/GCrat-Pit1-ChIP-Seq(GSE58009)/Homer | 1e-24 | -5.571e+01 | 0.0000 | 7739.0 | 36.72% | 4073.9 | 33.37% | motif file (matrix) | svg |
| 41 | T A G C G T A C C G T A C T A G A C T G T G C A C G T A A T G C C G T A A T C G | AR-halfsite(NR)/LNCaP-AR-ChIP-Seq(GSE27824)/Homer | 1e-23 | -5.433e+01 | 0.0000 | 16433.0 | 77.97% | 9156.0 | 75.00% | motif file (matrix) | svg |
| 42 | A T G C G A T C G A C T A G C T C G A T C G A T G T C A C G A T T C G A A T C G T A G C T A G C | TATA-Box(TBP)/Promoter/Homer | 1e-22 | -5.291e+01 | 0.0000 | 8535.0 | 40.50% | 4536.6 | 37.16% | motif file (matrix) | svg |
| 43 | G C T A A C G T C T A G G T C A C G T A A C G T C G T A C G A T C A G T A G T C C G T A A C G T C T A G C T G A A T C G | OCT:OCT(POU,Homeobox)/NPC-Brn1-ChIP-Seq(GSE35496)/Homer | 1e-21 | -4.974e+01 | 0.0000 | 147.0 | 0.70% | 34.8 | 0.28% | motif file (matrix) | svg |
| 44 | C A T G T G A C C G T A A G T C T A C G G C A T A C T G G T C A A T G C A G T C | bHLHE41(bHLH)/proB-Bhlhe41-ChIP-Seq(GSE93764)/Homer | 1e-20 | -4.690e+01 | 0.0000 | 4918.0 | 23.34% | 2525.0 | 20.68% | motif file (matrix) | svg |
| 45 | T C A G T C A G T A G C A G T C C T G A A G T C C T A G A C G T A C T G A T C G | c-Myc(bHLH)/mES-cMyc-ChIP-Seq(GSE11431)/Homer | 1e-19 | -4.578e+01 | 0.0000 | 2699.0 | 12.81% | 1316.8 | 10.79% | motif file (matrix) | svg |
| 46 | C A T G A C T G C T A G T C G A T C G A T C G A T C G A T C A G T C A G T C A G T G A C T G A C C G T A A C T G T G C A C G A T A C T G | RBPJ:Ebox(?,bHLH)/Panc1-Rbpj1-ChIP-Seq(GSE47459)/Homer | 1e-18 | -4.322e+01 | 0.0000 | 2507.0 | 11.90% | 1220.6 | 10.00% | motif file (matrix) | svg |
| 47 | T G A C G C T A T G A C C G T A T C A G G A T C C G T A C A T G C A T G C T A G C T A G C T A G | Unknown-ESC-element(?)/mES-Nanog-ChIP-Seq(GSE11724)/Homer | 1e-18 | -4.262e+01 | 0.0000 | 3340.0 | 15.85% | 1672.6 | 13.70% | motif file (matrix) | svg |
| 48 | C T A G C A G T T G A C C G T A G A T C T C A G G A C T C A T G | BMAL1(bHLH)/Liver-Bmal1-ChIP-Seq(GSE39860)/Homer | 1e-18 | -4.236e+01 | 0.0000 | 9722.0 | 46.13% | 5261.4 | 43.10% | motif file (matrix) | svg |
| 49 | G C T A C T G A T C G A A G T C A G T C C T G A A G T C G T C A C T G A T G C A | RUNX1(Runt)/Jurkat-RUNX1-ChIP-Seq(GSE29180)/Homer | 1e-18 | -4.185e+01 | 0.0000 | 6004.0 | 28.49% | 3151.0 | 25.81% | motif file (matrix) | svg |
| 50 | G A C T A G T C C G A T A C T G C T G A T G A C G T A C C G T A A T C G G C A T C T G A C T A G | Bcl11a(Zf)/HSPC-BCL11A-ChIP-Seq(GSE104676)/Homer | 1e-17 | -4.105e+01 | 0.0000 | 3296.0 | 15.64% | 1653.4 | 13.54% | motif file (matrix) | svg |
| 51 | T A G C T A G C G C A T C A T G A C T G G C T A C G T A A C G T A C T G G A T C | TEAD4(TEA)/Tropoblast-Tead4-ChIP-Seq(GSE37350)/Homer | 1e-17 | -3.969e+01 | 0.0000 | 4895.0 | 23.23% | 2539.6 | 20.80% | motif file (matrix) | svg |
| 52 | T C A G G A C T C A G T C T G A A G C T C T A G G A C T T G C A C T G A A G T C | HLF(bZIP)/HSC-HLF.Flag-ChIP-Seq(GSE69817)/Homer | 1e-16 | -3.877e+01 | 0.0000 | 6585.0 | 31.25% | 3490.6 | 28.59% | motif file (matrix) | svg |
| 53 | T C G A A C G T A C G T C T G A G A T C T C A G G A C T G T C A C G T A A G C T G T C A C T A G A G C T A C G T T C G A | NFIL3(bZIP)/HepG2-NFIL3-ChIP-Seq(Encode)/Homer | 1e-16 | -3.832e+01 | 0.0000 | 5253.0 | 24.93% | 2744.0 | 22.48% | motif file (matrix) | svg |
| 54 | T C G A T G A C A G T C C G T A A G T C C T A G A C G T A C T G A C T G A G C T A G T C G C A T | Max(bHLH)/K562-Max-ChIP-Seq(GSE31477)/Homer | 1e-16 | -3.792e+01 | 0.0000 | 3926.0 | 18.63% | 2009.8 | 16.46% | motif file (matrix) | svg |
| 55 | C T G A C T G A C T G A A T G C G A T C C A T G A C T G G A C T G A C T G C A T C G T A C G T A A G T C G T A C C T G A A T C G G C A T G A C T G A C T A G C T | GRHL2(CP2)/HBE-GRHL2-ChIP-Seq(GSE46194)/Homer | 1e-15 | -3.639e+01 | 0.0000 | 2354.0 | 11.17% | 1157.1 | 9.48% | motif file (matrix) | svg |
| 56 | A G T C G A T C A G T C C G T A A T C G C A G T A G T C G T A C C T G A A C T G T C A G A G C T A G C T A G C T A G C T | PRDM15(Zf)/ESC-Prdm15-ChIP-Seq(GSE73694)/Homer | 1e-15 | -3.632e+01 | 0.0000 | 5681.0 | 26.96% | 2992.3 | 24.51% | motif file (matrix) | svg |
| 57 | T G A C G C T A T C G A T G C A A G T C A G T C C G T A A G T C C G T A C T G A G C T A G T A C | RUNX2(Runt)/PCa-RUNX2-ChIP-Seq(GSE33889)/Homer | 1e-15 | -3.618e+01 | 0.0000 | 5060.0 | 24.01% | 2645.9 | 21.67% | motif file (matrix) | svg |
| 58 | C G A T T G C A G T A C C G T A A G T C C T A G G A C T C A T G | NPAS(bHLH)/Liver-NPAS-ChIP-Seq(GSE39860)/Homer | 1e-15 | -3.599e+01 | 0.0000 | 8738.0 | 41.46% | 4727.4 | 38.73% | motif file (matrix) | svg |
| 59 | G C A T T C A G C T G A A T C G A C T G C G A T G A T C C T G A | THRb(NR)/Liver-NR1A2-ChIP-Seq(GSE52613)/Homer | 1e-14 | -3.448e+01 | 0.0000 | 14275.0 | 67.73% | 7952.7 | 65.15% | motif file (matrix) | svg |
| 60 | C G A T T G C A T G C A G A T C C G T A A C T G T G A C G A C T C A T G A C T G | Tcf21(bHLH)/ArterySmoothMuscle-Tcf21-ChIP-Seq(GSE61369)/Homer | 1e-14 | -3.348e+01 | 0.0000 | 7176.0 | 34.05% | 3849.8 | 31.54% | motif file (matrix) | svg |
| 61 | T A C G T C G A T A G C A G T C C G T A A G T C C T A G G C A T A C T G A T C G | n-Myc(bHLH)/mES-nMyc-ChIP-Seq(GSE11431)/Homer | 1e-14 | -3.258e+01 | 0.0000 | 3480.0 | 16.51% | 1783.2 | 14.61% | motif file (matrix) | svg |
| 62 | C A T G T A C G T A G C G A T C G A T C A T G C G T A C G A C T T C A G A T G C C G A T A T C G C A G T A C T G G T A C | Zic3(Zf)/mES-Zic3-ChIP-Seq(GSE37889)/Homer | 1e-14 | -3.231e+01 | 0.0000 | 2702.0 | 12.82% | 1358.1 | 11.13% | motif file (matrix) | svg |
| 63 | T A G C T C A G C A T G G C A T A G C T C G A T A T G C C G T A C G T A G T C A | CHR(?)/Hela-CellCycle-Expression/Homer | 1e-13 | -3.206e+01 | 0.0000 | 4570.0 | 21.68% | 2389.9 | 19.58% | motif file (matrix) | svg |
| 64 | C G A T C T A G T C A G C A G T C G T A A G T C G C T A A C G T G A C T A T G C A G T C G C T A | PRDM10(Zf)/HEK293-PRDM10.eGFP-ChIP-Seq(Encode)/Homer | 1e-13 | -3.116e+01 | 0.0000 | 3325.0 | 15.78% | 1703.3 | 13.95% | motif file (matrix) | svg |
| 65 | A G C T A G C T C A T G C T G A G T A C A G T C A G C T A G C T C A G T C T A G | RARa(NR)/K562-RARa-ChIP-Seq(Encode)/Homer | 1e-13 | -3.094e+01 | 0.0000 | 11337.0 | 53.79% | 6252.2 | 51.22% | motif file (matrix) | svg |
| 66 | G T C A T G C A G C T A A G T C C G T A A C T G T G A C G C A T T C A G C A G T | Ap4(bHLH)/AML-Tfap4-ChIP-Seq(GSE45738)/Homer | 1e-13 | -3.081e+01 | 0.0000 | 8181.0 | 38.82% | 4435.7 | 36.34% | motif file (matrix) | svg |
| 67 | C G T A A C G T A C T G G T A C C G T A A C G T C G T A C G T A A C G T A C T G A G T C C G T A A C G T C T G A G C A T | OCT:OCT-short(POU,Homeobox)/NPC-OCT6-ChIP-Seq(GSE43916)/Homer | 1e-13 | -3.062e+01 | 0.0000 | 5468.0 | 25.95% | 2898.6 | 23.74% | motif file (matrix) | svg |
| 68 | A G C T C T A G T G A C C G T A A C G T C G A T A G T C A G T C C T G A C A T G | TEAD3(TEA)/HepG2-TEAD3-ChIP-Seq(Encode)/Homer | 1e-13 | -3.047e+01 | 0.0000 | 6864.0 | 32.57% | 3687.2 | 30.20% | motif file (matrix) | svg |
| 69 | G C T A A G C T T A C G G T C A C T G A C G A T C T G A G C A T C A G T A G T C | Brn2(POU,Homeobox)/NPC-Brn2-ChIP-Seq(GSE35496)/Homer | 1e-12 | -2.946e+01 | 0.0000 | 969.0 | 4.60% | 442.0 | 3.62% | motif file (matrix) | svg |
| 70 | C T A G C T A G C G T A C G T A T A C G C G A T C T A G C T G A C T G A C G T A T A C G G A C T | PU.1:IRF8(ETS:IRF)/pDC-Irf8-ChIP-Seq(GSE66899)/Homer | 1e-12 | -2.924e+01 | 0.0000 | 1245.0 | 5.91% | 586.9 | 4.81% | motif file (matrix) | svg |
| 71 | C T G A T C A G G T A C T G C A A G T C C G T A A G T C A C T G A C G T A C T G | MNT(bHLH)/HepG2-MNT-ChIP-Seq(Encode)/Homer | 1e-12 | -2.810e+01 | 0.0000 | 6131.0 | 29.09% | 3284.8 | 26.91% | motif file (matrix) | svg |
| 72 | T G A C C T A G T C A G G T C A C G T A T C A G C G A T T C A G T C G A T G C A C T G A T A G C | PU.1-IRF(ETS:IRF)/Bcell-PU.1-ChIP-Seq(GSE21512)/Homer | 1e-11 | -2.678e+01 | 0.0000 | 7899.0 | 37.48% | 4296.7 | 35.20% | motif file (matrix) | svg |
| 73 | G A C T C A G T G A T C G A T C A C G T G A T C C T G A T A C G C G T A G T C A | STAT6(Stat)/Macrophage-Stat6-ChIP-Seq(GSE38377)/Homer | 1e-11 | -2.654e+01 | 0.0000 | 4017.0 | 19.06% | 2105.8 | 17.25% | motif file (matrix) | svg |
| 74 | T G A C T C G A C T G A C T G A A T G C G A T C C T A G T A C G G A C T G A C T G A T C T C G A C T G A C T G A A T G C G A T C C T A G A T C G G A C T G A C T | Tcfcp2l1(CP2)/mES-Tcfcp2l1-ChIP-Seq(GSE11431)/Homer | 1e-10 | -2.533e+01 | 0.0000 | 772.0 | 3.66% | 349.8 | 2.87% | motif file (matrix) | svg |
| 75 | C T G A T C A G A G T C C G T A A T C G A T G C C G A T A C T G A G T C G A C T A T C G A G T C | MyoD(bHLH)/Myotube-MyoD-ChIP-Seq(GSE21614)/Homer | 1e-10 | -2.521e+01 | 0.0000 | 5213.0 | 24.74% | 2781.0 | 22.78% | motif file (matrix) | svg |
| 76 | A C T G G A T C G A C T A C T G A C G T C A T G A C T G A C G T A G C T C G A T | RUNX-AML(Runt)/CD4+-PolII-ChIP-Seq(Barski\_et\_al.)/Homer | 1e-10 | -2.506e+01 | 0.0000 | 4263.0 | 20.23% | 2249.1 | 18.42% | motif file (matrix) | svg |
| 77 | G A C T C G A T T C A G G A T C G A C T A G C T A G C T A G T C G A T C C G T A C T A G C T A G T C G A T C G A C T G A | Bcl6(Zf)/Liver-Bcl6-ChIP-Seq(GSE31578)/Homer | 1e-10 | -2.457e+01 | 0.0000 | 8249.0 | 39.14% | 4509.8 | 36.94% | motif file (matrix) | svg |
| 78 | C T G A A G C T A C G T A C G T A G T C G A C T G A C T C T G A C T G A C T A G C G T A C G T A | STAT6(Stat)/CD4-Stat6-ChIP-Seq(GSE22104)/Homer | 1e-10 | -2.437e+01 | 0.0000 | 3958.0 | 18.78% | 2082.2 | 17.06% | motif file (matrix) | svg |
| 79 | T G A C G T C A A G T C G C T A C T A G A G T C C T G A C A T G A C T G C T A G T A C G T C A G | Zic2(Zf)/ESC-Zic2-ChIP-Seq(SRP197560)/Homer | 1e-10 | -2.386e+01 | 0.0000 | 2094.0 | 9.94% | 1056.9 | 8.66% | motif file (matrix) | svg |
| 80 | G C T A A G T C T A C G T G C A A T C G T C A G G C T A T C G A T C A G A G C T | ELF5(ETS)/T47D-ELF5-ChIP-Seq(GSE30407)/Homer | 1e-10 | -2.336e+01 | 0.0000 | 4352.0 | 20.65% | 2307.3 | 18.90% | motif file (matrix) | svg |
| 81 | T A G C G C T A T C G A C T G A A G T C A G T C C T G A A G T C C G T A C T A G | RUNX(Runt)/HPC7-Runx1-ChIP-Seq(GSE22178)/Homer | 1e-9 | -2.218e+01 | 0.0000 | 4027.0 | 19.11% | 2131.3 | 17.46% | motif file (matrix) | svg |
| 82 | C G A T C T A G C T G A A T G C C T G A T C G A C G T A C T G A T C G A T A G C A G T C C G T A A C T G T C G A A T G C | Hand2(bHLH)/Mesoderm-Hand2-ChIP-Seq(GSE61475)/Homer | 1e-9 | -2.207e+01 | 0.0000 | 3135.0 | 14.88% | 1635.4 | 13.40% | motif file (matrix) | svg |
| 83 | C G A T C T G A C G T A C A G T A G T C G A T C G A T C A C T G | Pitx1(Homeobox)/Chicken-Pitx1-ChIP-Seq(GSE38910)/Homer | 1e-9 | -2.197e+01 | 0.0000 | 17127.0 | 81.27% | 9712.1 | 79.56% | motif file (matrix) | svg |
| 84 | C A G T A C T G T C A G T G C A G C T A A T G C T C G A A T C G G T C A T G C A | ZNF189(Zf)/HEK293-ZNF189.GFP-ChIP-Seq(GSE58341)/Homer | 1e-9 | -2.137e+01 | 0.0000 | 4930.0 | 23.39% | 2643.0 | 21.65% | motif file (matrix) | svg |
| 85 | T G A C C G A T C T G A C T A G C T A G A C G T A T G C T G C A T C G A C T G A C T A G C A T G A C G T A G T C C G T A | PPARa(NR),DR1/Liver-Ppara-ChIP-Seq(GSE47954)/Homer | 1e-9 | -2.093e+01 | 0.0000 | 4572.0 | 21.69% | 2443.6 | 20.02% | motif file (matrix) | svg |
| 86 | C G T A C G T A C G T A A G T C A G C T C T G A A C T G C T A G A G C T A G T C C G T A T C A G | RORg(NR)/Liver-Rorc-ChIP-Seq(GSE101115)/Homer | 1e-8 | -2.030e+01 | 0.0000 | 688.0 | 3.26% | 316.7 | 2.59% | motif file (matrix) | svg |
| 87 | G T C A T C G A C T A G C T A G A G T C G T C A C G A T C T A G G A C T G A T C G A T C T C A G C T A G C T G A A G T C G C T A C A G T T C A G G A T C G A T C | p63(p53)/Keratinocyte-p63-ChIP-Seq(GSE17611)/Homer | 1e-8 | -2.030e+01 | 0.0000 | 1975.0 | 9.37% | 1004.3 | 8.23% | motif file (matrix) | svg |
| 88 | G A T C G T A C C G A T A C T G A C T G C G T A C G T A A C G T A C T G G A T C | TEAD(TEA)/Fibroblast-PU.1-ChIP-Seq(Unpublished)/Homer | 1e-8 | -2.019e+01 | 0.0000 | 4182.0 | 19.84% | 2228.4 | 18.25% | motif file (matrix) | svg |
| 89 | G T C A A C G T C T A G G T C A C G T A G C A T C G T A C G T A G C A T C A G T A G T C C G T A C A G T C T A G C T G A | OCT:OCT(POU,Homeobox,IR1)/NPC-Brn2-ChIP-Seq(GSE35496)/Homer | 1e-8 | -1.971e+01 | 0.0000 | 197.0 | 0.93% | 73.0 | 0.60% | motif file (matrix) | svg |
| 90 | C G T A C G T A C G T A A G T C A G C T C T G A A C T G A C T G A G C T A G T C C G T A C T A G C T A G C T A G T G C A | RORa(NR)/Liver-Rora-ChIP-Seq(GSE101115)/Homer | 1e-8 | -1.956e+01 | 0.0000 | 962.0 | 4.56% | 461.7 | 3.78% | motif file (matrix) | svg |
| 91 | A T C G G A T C G C A T C G T A G T C A A C G T A T G C A G T C | CRX(Homeobox)/Retina-Crx-ChIP-Seq(GSE20012)/Homer | 1e-8 | -1.918e+01 | 0.0000 | 11805.0 | 56.01% | 6598.0 | 54.05% | motif file (matrix) | svg |
| 92 | T C G A T A G C T G C A A C T G A C T G C G T A C G T A C T A G G A C T T A C G | ETS1(ETS)/Jurkat-ETS1-ChIP-Seq(GSE17954)/Homer | 1e-8 | -1.912e+01 | 0.0000 | 5622.0 | 26.68% | 3046.5 | 24.96% | motif file (matrix) | svg |
| 93 | T C G A A C T G A C T G C G T A C G T A T C G A A G T C C T G A A T C G G T A C G C A T C A T G | ETS:E-box(ETS,bHLH)/HPC7-Scl-ChIP-Seq(GSE22178)/Homer | 1e-8 | -1.895e+01 | 0.0000 | 868.0 | 4.12% | 413.8 | 3.39% | motif file (matrix) | svg |
| 94 | C T A G C A T G C A T G A G C T G A C T C G T A G A T C A G C T G T C A A T G C T C G A C T A G C A T G A C G T A G T C C T G A | LXRE(NR),DR4/RAW-LXRb.biotin-ChIP-Seq(GSE21512)/Homer | 1e-8 | -1.845e+01 | 0.0000 | 222.0 | 1.05% | 86.7 | 0.71% | motif file (matrix) | svg |
| 95 | T G A C A G T C C T G A T G A C C G T A A C G T A C G T A G T C A G T C C G T A | TEAD1(TEAD)/HepG2-TEAD1-ChIP-Seq(Encode)/Homer | 1e-7 | -1.835e+01 | 0.0000 | 5730.0 | 27.19% | 3112.8 | 25.50% | motif file (matrix) | svg |
| 96 | T C G A C T A G A G T C A G T C C G T A C G T A A C G T T A G C T C A G T A C G | NFY(CCAAT)/Promoter/Homer | 1e-7 | -1.747e+01 | 0.0000 | 3833.0 | 18.19% | 2047.4 | 16.77% | motif file (matrix) | svg |
| 97 | G C T A G C A T G A C T G C A T T C A G G T A C G C T A G C A T C T G A G C T A T A G C G C T A C T G A C G A T C T A G | OCT4-SOX2-TCF-NANOG(POU,Homeobox,HMG)/mES-Oct4-ChIP-Seq(GSE11431)/Homer | 1e-7 | -1.726e+01 | 0.0000 | 1602.0 | 7.60% | 812.4 | 6.65% | motif file (matrix) | svg |
| 98 | A G T C G A C T C A G T G T A C A G T C A T C G T C A G A C T G G T C A C G T A | Stat3(Stat)/mES-Stat3-ChIP-Seq(GSE11431)/Homer | 1e-7 | -1.682e+01 | 0.0000 | 2959.0 | 14.04% | 1562.4 | 12.80% | motif file (matrix) | svg |
| 99 | C T A G C G T A G T C A C G T A A G T C G A T C A G C T C T A G C G T A A C G T G T C A G A T C | Six2(Homeobox)/NephronProgenitor-Six2-ChIP-Seq(GSE39837)/Homer | 1e-7 | -1.656e+01 | 0.0000 | 5919.0 | 28.09% | 3231.7 | 26.47% | motif file (matrix) | svg |
| 100 | A G T C G A C T A C T G G A T C G T A C C G T A T G A C A G T C C G A T A G C T A C G T A C G T C T A G G A C T C T G A | ZNF7(Zf)/HepG2-ZNF7.Flag-ChIP-Seq(Encode)/Homer | 1e-7 | -1.625e+01 | 0.0000 | 3794.0 | 18.00% | 2033.0 | 16.65% | motif file (matrix) | svg |
| 101 | G A C T A G C T G T A C G A C T C T G A A C T G G T C A C T G A A T G C T A C G G A C T A C G T A G T C G A C T C T G A | HRE(HSF)/Striatum-HSF1-ChIP-Seq(GSE38000)/Homer | 1e-6 | -1.584e+01 | 0.0000 | 1506.0 | 7.15% | 765.8 | 6.27% | motif file (matrix) | svg |
| 102 | G C T A C G T A A G T C A C G T T C G A T A C G A C T G A G C T A G T C T C G A | RORgt(NR)/EL4-RORgt.Flag-ChIP-Seq(GSE56019)/Homer | 1e-6 | -1.535e+01 | 0.0000 | 865.0 | 4.10% | 421.1 | 3.45% | motif file (matrix) | svg |
| 103 | G C T A C G T A A G T C A C G T T C G A T A C G A C T G A G C T A G T C T C G A | RORgt(NR)/EL4-RORgt.Flag-ChIP-Seq(GSE56019)/Homer | 1e-6 | -1.535e+01 | 0.0000 | 865.0 | 4.10% | 421.1 | 3.45% | motif file (matrix) | svg |
| 104 | C G T A A C G T A C G T A C G T A C G T A G T C A G T C C T G A A G C T A G C T | NFAT(RHD)/Jurkat-NFATC1-ChIP-Seq(Jolma\_et\_al.)/Homer | 1e-6 | -1.530e+01 | 0.0000 | 5916.0 | 28.07% | 3238.9 | 26.53% | motif file (matrix) | svg |
| 105 | T C A G A G C T A T G C C G T A A G C T T C A G C A G T A C T G C T G A A G T C | MITF(bHLH)/MastCells-MITF-ChIP-Seq(GSE48085)/Homer | 1e-6 | -1.493e+01 | 0.0000 | 4773.0 | 22.65% | 2592.3 | 21.24% | motif file (matrix) | svg |
| 106 | C G T A C T G A A C T G A G C T A G T C G A T C G A T C G C A T C T G A C T A G C T A G T A C G T C G A T G C A G C A T | EBF2(EBF)/BrownAdipose-EBF2-ChIP-Seq(GSE97114)/Homer | 1e-6 | -1.449e+01 | 0.0000 | 4276.0 | 20.29% | 2314.3 | 18.96% | motif file (matrix) | svg |
| 107 | G C T A T A G C A G C T A T C G G T C A C G T A G C T A A T G C G A T C C T G A | IRF4(IRF)/GM12878-IRF4-ChIP-Seq(GSE32465)/Homer | 1e-6 | -1.431e+01 | 0.0000 | 2661.0 | 12.63% | 1409.9 | 11.55% | motif file (matrix) | svg |
| 108 | T G C A G C A T C G A T C G T A C A G T A C T G G T A C C G T A C T G A A G C T G T C A A C T G C T A G G T C A C G A T A C T G G T A C T G C A C G T A A G C T | CEBP:CEBP(bZIP)/MEF-Chop-ChIP-Seq(GSE35681)/Homer | 1e-6 | -1.431e+01 | 0.0000 | 1068.0 | 5.07% | 533.3 | 4.37% | motif file (matrix) | svg |
| 109 | C G A T G A C T C G A T T C A G G A C T A C G T C A G T C T G A G A C T G A C T A G C T C G A T A C T G A T C G G T A C G C T A | NF1:FOXA1(CTF,Forkhead)/LNCAP-FOXA1-ChIP-Seq(GSE27824)/Homer | 1e-5 | -1.356e+01 | 0.0000 | 428.0 | 2.03% | 197.0 | 1.61% | motif file (matrix) | svg |
| 110 | C T G A T A C G G C A T A G C T A G C T A G T C T C G A A C T G C A G T A G C T A G C T G A T C | IRF3(IRF)/BMDM-Irf3-ChIP-Seq(GSE67343)/Homer | 1e-5 | -1.316e+01 | 0.0000 | 2334.0 | 11.07% | 1233.2 | 10.10% | motif file (matrix) | svg |
| 111 | C A T G G A C T G C A T A C T G A G C T A C T G A C T G C G T A G C A T A G C T A T C G T A C G | Foxh1(Forkhead)/hESC-FOXH1-ChIP-Seq(GSE29422)/Homer | 1e-5 | -1.298e+01 | 0.0000 | 3897.0 | 18.49% | 2110.8 | 17.29% | motif file (matrix) | svg |
| 112 | A G T C T G C A T C G A C T G A A C T G C A T G A C G T A T G C G T C A T A C G | Erra(NR)/HepG2-Erra-ChIP-Seq(GSE31477)/Homer | 1e-5 | -1.243e+01 | 0.0000 | 8398.0 | 39.85% | 4681.1 | 38.35% | motif file (matrix) | svg |
| 113 | T C G A T G A C G C A T A G C T C A G T G A T C G C T A G A T C G A C T A C G T G C A T A G T C | PRDM1(Zf)/Hela-PRDM1-ChIP-Seq(GSE31477)/Homer | 1e-5 | -1.225e+01 | 0.0000 | 3523.0 | 16.72% | 1904.3 | 15.60% | motif file (matrix) | svg |
| 114 | C G A T T A C G T G C A G T A C G A T C G A C T A G C T A C G T A T C G G T A C G A T C G T A C G A T C G T C A | PPARE(NR),DR1/3T3L1-Pparg-ChIP-Seq(GSE13511)/Homer | 1e-5 | -1.202e+01 | 0.0000 | 4029.0 | 19.12% | 2191.9 | 17.96% | motif file (matrix) | svg |
| 115 | C A T G G A C T T A C G G T C A G T A C G A T C G A C T A G C T A T C G T C G A T A C G T A G C | ERRg(NR)/Kidney-ESRRG-ChIP-Seq(GSE104905)/Homer | 1e-5 | -1.158e+01 | 0.0000 | 3469.0 | 16.46% | 1878.7 | 15.39% | motif file (matrix) | svg |
| 116 | T C A G T C A G G C T A C G T A T A C G G A C T T C A G T C G A C T G A C G T A T A C G G A C T | IRF8(IRF)/BMDM-IRF8-ChIP-Seq(GSE77884)/Homer | 1e-4 | -1.132e+01 | 0.0000 | 2045.0 | 9.70% | 1082.8 | 8.87% | motif file (matrix) | svg |
| 117 | C T G A T A G C G C T A C G A T A T C G A G T C G A T C G A T C C T A G T C A G T C A G G T A C G C T A C A G T | p53(p53)/mES-cMyc-ChIP-Seq(GSE11431)/Homer | 1e-4 | -1.132e+01 | 0.0000 | 74.0 | 0.35% | 25.3 | 0.21% | motif file (matrix) | svg |
| 118 | A G T C G T A C A G C T C T A G A G T C C G A T A C T G C G T A A C T G G T C A | Zic(Zf)/Cerebellum-ZIC1.2-ChIP-Seq(GSE60731)/Homer | 1e-4 | -1.119e+01 | 0.0001 | 4578.0 | 21.72% | 2508.4 | 20.55% | motif file (matrix) | svg |
| 119 | T G C A C T G A A T G C G T C A A C T G A C T G C G T A C G T A C T A G A G C T | Ets1-distal(ETS)/CD4+-PolII-ChIP-Seq(Barski\_et\_al.)/Homer | 1e-4 | -1.096e+01 | 0.0001 | 1957.0 | 9.29% | 1035.0 | 8.48% | motif file (matrix) | svg |
| 120 | A G T C G A T C G C T A C G A T A C G T T A C G G C A T C T G A G A C T A C T G A G T C G C T A C T G A T C G A C A G T | Oct4:Sox17(POU,Homeobox,HMG)/F9-Sox17-ChIP-Seq(GSE44553)/Homer | 1e-4 | -1.064e+01 | 0.0001 | 1040.0 | 4.93% | 531.3 | 4.35% | motif file (matrix) | svg |
| 121 | A T G C A G T C C T G A A G T C C G A T A C G T A G T C A G T C A C G T A T C G G A C T A C G T | Etv2(ETS)/ES-ER71-ChIP-Seq(GSE59402)/Homer | 1e-4 | -1.037e+01 | 0.0001 | 5226.0 | 24.80% | 2883.1 | 23.62% | motif file (matrix) | svg |
| 122 | A T G C T A G C A G C T A G C T T G A C G A C T T C A G T A C G G T C A C T G A A T C G T A G C G A C T C A G T A G T C A G C T T C G A A T C G T G C A T G C A | HRE(HSF)/HepG2-HSF1-ChIP-Seq(GSE31477)/Homer | 1e-4 | -9.865e+00 | 0.0002 | 871.0 | 4.13% | 443.0 | 3.63% | motif file (matrix) | svg |
| 123 | C T G A A T G C C G T A A C G T A G T C A G T C A C G T A C T G A T C G G C A T | SPDEF(ETS)/VCaP-SPDEF-ChIP-Seq(SRA014231)/Homer | 1e-4 | -9.530e+00 | 0.0003 | 5655.0 | 26.83% | 3135.4 | 25.68% | motif file (matrix) | svg |
| 124 | C T G A C G A T C T A G T C A G G A T C C T G A T C A G G A T C C T G A A C T G A G T C G C T A A C G T A G T C G C A T | PRDM9(Zf)/Testis-DMC1-ChIP-Seq(GSE35498)/Homer | 1e-3 | -9.197e+00 | 0.0004 | 2760.0 | 13.10% | 1495.3 | 12.25% | motif file (matrix) | svg |
| 125 | T C A G C T A G C T A G T G C A C G A T C G A T C G T A C T A G | GSC(Homeobox)/FrogEmbryos-GSC-ChIP-Seq(DRA000576)/Homer | 1e-3 | -9.182e+00 | 0.0004 | 6773.0 | 32.14% | 3778.1 | 30.95% | motif file (matrix) | svg |
| 126 | A G C T C T G A C T A G C T A G A C T G T A G C T G C A T C G A C T G A C T A G C A T G A C G T A T G C T C G A | RXR(NR),DR1/3T3L1-RXR-ChIP-Seq(GSE13511)/Homer | 1e-3 | -8.711e+00 | 0.0006 | 4602.0 | 21.84% | 2542.2 | 20.83% | motif file (matrix) | svg |
| 127 | G A T C C T G A A G T C C G A T C G A T G A T C A G T C A C T G A T C G A G C T | Elk4(ETS)/Hela-Elk4-ChIP-Seq(GSE31477)/Homer | 1e-3 | -8.509e+00 | 0.0007 | 2216.0 | 10.51% | 1194.1 | 9.78% | motif file (matrix) | svg |
| 128 | T C A G C T G A C G T A C G T A T A C G G C A T C T A G C T G A C G T A C G T A T A C G G A C T | IRF1(IRF)/PBMC-IRF1-ChIP-Seq(GSE43036)/Homer | 1e-3 | -8.342e+00 | 0.0008 | 924.0 | 4.38% | 477.0 | 3.91% | motif file (matrix) | svg |
| 129 | C T G A T C A G A C T G C A G T A G T C G A C T A G T C A C G T C G T A G C T A A T G C G A T C | PRDM14(Zf)/H1-PRDM14-ChIP-Seq(GSE22767)/Homer | 1e-3 | -8.172e+00 | 0.0010 | 1701.0 | 8.07% | 908.2 | 7.44% | motif file (matrix) | svg |
| 130 | T G C A C T G A C A T G C T A G C A G T A G T C C G T A A T G C A T G C T A C G G C A T T C A G G T C A G A T C G T A C | ERE(NR),IR3/MCF7-ERa-ChIP-Seq(Unpublished)/Homer | 1e-3 | -7.763e+00 | 0.0014 | 1195.0 | 5.67% | 630.0 | 5.16% | motif file (matrix) | svg |
| 131 | T C G A T A G C G T C A A C T G A C T G C G T A C G T A C T A G A G C T T C A G | ERG(ETS)/VCaP-ERG-ChIP-Seq(GSE14097)/Homer | 1e-3 | -7.722e+00 | 0.0015 | 8423.0 | 39.97% | 4742.8 | 38.85% | motif file (matrix) | svg |
| 132 | C T A G T C G A C G A T C T A G G C A T C A G T C T A G G A T C C G T A G T C A | CEBP:AP1(bZIP)/ThioMac-CEBPb-ChIP-Seq(GSE21512)/Homer | 1e-3 | -7.688e+00 | 0.0015 | 4959.0 | 23.53% | 2755.4 | 22.57% | motif file (matrix) | svg |
| 133 | A C T G A G C T A G T C G T C A A G C T T C A G A T G C G A T C G C A T A T C G T C G A T A G C C G A T C A T G T A G C | Pax8(Paired,Homeobox)/Thyroid-Pax8-ChIP-Seq(GSE26938)/Homer | 1e-3 | -7.655e+00 | 0.0016 | 1579.0 | 7.49% | 843.7 | 6.91% | motif file (matrix) | svg |
| 134 | A T C G G A C T C G A T C G T A C G T A C A G T G A T C G A T C G A T C A G C T | Otx2(Homeobox)/EpiLC-Otx2-ChIP-Seq(GSE56098)/Homer | 1e-3 | -7.610e+00 | 0.0016 | 4586.0 | 21.76% | 2543.4 | 20.83% | motif file (matrix) | svg |
| 135 | T A G C C G T A C T G A T A C G C G T A A C G T A C T G A C T G A G T C T A C G C T A G G T A C | YY1(Zf)/Promoter/Homer | 1e-3 | -7.392e+00 | 0.0020 | 215.0 | 1.02% | 99.2 | 0.81% | motif file (matrix) | svg |
| 136 | G T A C G C T A T C A G C T G A C T A G C A T G A G C T G A T C T G C A T C G A C T G A A C T G C A G T A G T C G A T C G C T A | HNF4a(NR),DR1/HepG2-HNF4a-ChIP-Seq(GSE25021)/Homer | 1e-3 | -7.054e+00 | 0.0028 | 2136.0 | 10.14% | 1159.4 | 9.50% | motif file (matrix) | svg |
| 137 | G C T A C G A T C T A G G T A C G C T A C G A T C G T A G C T A C G A T C A G T G A T C G C T A | Pit1+1bp(Homeobox)/GCrat-Pit1-ChIP-Seq(GSE58009)/Homer | 1e-3 | -6.932e+00 | 0.0031 | 2822.0 | 13.39% | 1547.0 | 12.67% | motif file (matrix) | svg |
| 138 | C G T A C T G A C T A G C T G A A G T C G C T A C G A T A T C G G A C T G A T C A G T C C T G A C T A G C T A G A G T C G C T A C G A T C T A G G A T C G A T C | p73(p53)/Trachea-p73-ChIP-Seq(PRJNA310161)/Homer | 1e-2 | -6.888e+00 | 0.0033 | 236.0 | 1.12% | 111.0 | 0.91% | motif file (matrix) | svg |
| 139 | T C G A A G C T A C G T A C G T A G T C A G T C A C G T A T C G G A C T A T C G | EWS:ERG-fusion(ETS)/CADO\_ES1-EWS:ERG-ChIP-Seq(SRA014231)/Homer | 1e-2 | -6.865e+00 | 0.0033 | 4645.0 | 22.04% | 2584.0 | 21.17% | motif file (matrix) | svg |
| 140 | C T G A T A G C T G A C T C A G C T A G G T C A C G T A T C A G A G C T T C A G | ETV4(ETS)/HepG2-ETV4-ChIP-Seq(ENCODE)/Homer | 1e-2 | -6.748e+00 | 0.0037 | 5119.0 | 24.29% | 2856.1 | 23.40% | motif file (matrix) | svg |
| 141 | C T G A T C A G G C T A A G C T A G T C G A C T C T G A C T A G T G C A C T G A A G T C G T A C G A T C A C T G T C G A | ZBTB12(Zf)/HEK293-ZBTB12.GFP-ChIP-Seq(GSE58341)/Homer | 1e-2 | -6.609e+00 | 0.0042 | 2203.0 | 10.45% | 1200.0 | 9.83% | motif file (matrix) | svg |
| 142 | A T G C A G T C G C T A C G A T C G T A G C A T G C T A C G A T C T A G C A T G T G A C G T C A | CArG(MADS)/PUER-Srf-ChIP-Seq(Sullivan\_et\_al.)/Homer | 1e-2 | -6.593e+00 | 0.0042 | 1979.0 | 9.39% | 1074.2 | 8.80% | motif file (matrix) | svg |
| 143 | A G T C C T G A A G T C C G A T C A G T G A T C A T G C A C T G A T C G G A C T | Fli1(ETS)/CD8-FLI-ChIP-Seq(GSE20898)/Homer | 1e-2 | -6.562e+00 | 0.0043 | 5479.0 | 26.00% | 3064.1 | 25.10% | motif file (matrix) | svg |
| 144 | T C G A T C G A T A G C G T A C T C A G T A C G C G T A C G T A T C A G A G C T | GABPA(ETS)/Jurkat-GABPa-ChIP-Seq(GSE17954)/Homer | 1e-2 | -6.499e+00 | 0.0046 | 4507.0 | 21.39% | 2509.1 | 20.55% | motif file (matrix) | svg |
| 145 | A T G C T C G A A G T C A G C T A C G T G T A C A G T C G C T A C T A G C A T G G T C A C T G A T C A G A G T C | Stat3+il21(Stat)/CD4-Stat3-ChIP-Seq(GSE19198)/Homer | 1e-2 | -6.412e+00 | 0.0050 | 4367.0 | 20.72% | 2430.9 | 19.91% | motif file (matrix) | svg |
| 146 | G C A T A T C G C A T G G T A C G C T A A G T C T C A G T G A C G T C A T G C A | Arnt:Ahr(bHLH)/MCF7-Arnt-ChIP-Seq(Lo\_et\_al.)/Homer | 1e-2 | -5.990e+00 | 0.0075 | 2910.0 | 13.81% | 1605.7 | 13.15% | motif file (matrix) | svg |
| 147 | C T A G A C T G T G C A G T C A A T G C C G T A A T C G A T G C A G T C C T A G | ZNF341(Zf)/EBV-ZNF341-ChIP-Seq(GSE113194)/Homer | 1e-2 | -5.979e+00 | 0.0076 | 4019.0 | 19.07% | 2236.1 | 18.32% | motif file (matrix) | svg |
| 148 | A C T G G A T C C T G A A T C G A G T C T A G C C T G A C G T A T A C G A G T C C T A G C A G T T C A G T C G A T G A C G A T C | PAX5(Paired,Homeobox)/GM12878-PAX5-ChIP-Seq(GSE32465)/Homer | 1e-2 | -5.943e+00 | 0.0078 | 1695.0 | 8.04% | 919.0 | 7.53% | motif file (matrix) | svg |
| 149 | T G C A C T G A A G T C G T C A A C T G A C T G C G T A C G T A C T G A A G C T | EWS:FLI1-fusion(ETS)/SK\_N\_MC-EWS:FLI1-ChIP-Seq(SRA014231)/Homer | 1e-2 | -5.891e+00 | 0.0082 | 3206.0 | 15.21% | 1774.6 | 14.54% | motif file (matrix) | svg |
| 150 | A T G C A G T C A G C T A G C T A C G T A T C G C G T A C G A T T A G C G A C T | LEF1(HMG)/H1-LEF1-ChIP-Seq(GSE64758)/Homer | 1e-2 | -5.641e+00 | 0.0104 | 4335.0 | 20.57% | 2420.7 | 19.83% | motif file (matrix) | svg |
| 151 | C T A G T A C G A G T C C G T A A G T C A C G T A G T C T C G A C G T A T A C G | Nkx2.1(Homeobox)/LungAC-Nkx2.1-ChIP-Seq(GSE43252)/Homer | 1e-2 | -5.626e+00 | 0.0105 | 13161.0 | 62.45% | 7513.9 | 61.55% | motif file (matrix) | svg |
| 152 | C T A G T A C G G A T C G T C A T C G A A C G T T C A G C G T A C G T A C G T A | Hoxd10(Homeobox)/ChickenMSG-Hoxd10.Flag-ChIP-Seq(GSE86088)/Homer | 1e-2 | -5.588e+00 | 0.0108 | 7082.0 | 33.60% | 3996.3 | 32.74% | motif file (matrix) | svg |
| 153 | C T A G C T G A A G T C G C T A C G A T A C T G G A C T G A T C G A T C C T G A C T A G C T G A T G A C G C T A C G A T T C A G G A C T G A T C G A T C T G A C | p53(p53)/Saos-p53-ChIP-Seq(GSE15780)/Homer | 1e-2 | -5.576e+00 | 0.0108 | 563.0 | 2.67% | 291.5 | 2.39% | motif file (matrix) | svg |
| 154 | C T A G C T G A A G T C G C T A C G A T A C T G G A C T G A T C G A T C C T G A C T A G C T G A T G A C G C T A C G A T T C A G G A C T G A T C G A T C T G A C | p53(p53)/Saos-p53-ChIP-Seq/Homer | 1e-2 | -5.576e+00 | 0.0108 | 563.0 | 2.67% | 291.5 | 2.39% | motif file (matrix) | svg |
| 155 | G A C T C A G T A G C T C G A T A G T C G A T C A G T C C G T A A T G C T C A G | Rbpj1(?)/Panc1-Rbpj1-ChIP-Seq(GSE47459)/Homer | 1e-2 | -5.554e+00 | 0.0110 | 8487.0 | 40.27% | 4806.8 | 39.38% | motif file (matrix) | svg |
| 156 | C G T A C T G A C G T A C T A G T C G A C T A G A C T G C G T A C G T A T A C G A G C T A T C G | SpiB(ETS)/OCILY3-SPIB-ChIP-Seq(GSE56857)/Homer | 1e-2 | -5.553e+00 | 0.0110 | 1373.0 | 6.51% | 741.1 | 6.07% | motif file (matrix) | svg |
| 157 | C G T A T G A C T A G C T G C A A C T G A C T G C G T A C G T A T C A G G A C T | ELF3(ETS)/PDAC-ELF3-ChIP-Seq(GSE64557)/Homer | 1e-2 | -5.503e+00 | 0.0114 | 4509.0 | 21.40% | 2521.1 | 20.65% | motif file (matrix) | svg |
| 158 | T A G C G A T C A G C T T G A C G C T A A G C T C A T G A C T G A C G T T C A G A G T C G A T C G A C T A G C T G C T A A G T C A G C T A G T C G A T C A T G C A G C T G A C T C A T G A C G T A T C G | ZNF41(Zf)/HEK293-ZNF41.GFP-ChIP-Seq(GSE58341)/Homer | 1e-2 | -5.424e+00 | 0.0123 | 151.0 | 0.72% | 70.5 | 0.58% | motif file (matrix) | svg |
| 159 | T C G A C T G A T A G C T G A C T C A G T C A G C G T A C G T A T C A G A G C T | ETV1(ETS)/GIST48-ETV1-ChIP-Seq(GSE22441)/Homer | 1e-2 | -5.329e+00 | 0.0134 | 6930.0 | 32.88% | 3912.8 | 32.05% | motif file (matrix) | svg |
| 160 | C G T A C A T G C A T G A C T G C T A G T C G A G C A T C G A T A G C T A G T C G A T C G T A C | NFkB-p65(RHD)/GM12787-p65-ChIP-Seq(GSE19485)/Homer | 1e-2 | -5.284e+00 | 0.0140 | 2449.0 | 11.62% | 1350.6 | 11.06% | motif file (matrix) | svg |
| 161 | C A T G A G C T T A C G G T C A G T A C T A G C A G C T G A C T A T C G T C G A | Esrrb(NR)/mES-Esrrb-ChIP-Seq(GSE11431)/Homer | 1e-2 | -4.977e+00 | 0.0188 | 2761.0 | 13.10% | 1530.3 | 12.54% | motif file (matrix) | svg |
| 162 | C T G A C T A G A T C G G C A T A C T G G T A C A T G C C G T A A C T G G C T A A G T C C G T A | Tbox:Smad(T-box,MAD)/ESCd5-Smad2\_3-ChIP-Seq(GSE29422)/Homer | 1e-2 | -4.778e+00 | 0.0229 | 1171.0 | 5.56% | 633.9 | 5.19% | motif file (matrix) | svg |
| 163 | G A C T T C G A C G A T T A C G C T A G T A C G A C T G A T G C G T A C G T A C | Zac1(Zf)/Neuro2A-Plagl1-ChIP-Seq(GSE75942)/Homer | 1e-2 | -4.713e+00 | 0.0242 | 9527.0 | 45.21% | 5419.9 | 44.40% | motif file (matrix) | svg |
| 164 | T G C A T G C A A G T C A G T C G A C T C A G T A T G C G A T C C T G A A C G T C T A G C T A G A G T C A C G T A G T C A G T C A G T C G A C T C G T A A C G T A G C T C T A G G A T C G A T C G A T C | ZNF16(Zf)/HEK293-ZNF16.GFP-ChIP-Seq(GSE58341)/Homer | 1e-2 | -4.642e+00 | 0.0259 | 39.0 | 0.19% | 15.8 | 0.13% | motif file (matrix) | svg |
| 165 | C T G A T C A G C T G A C T A G C A T G A C G T A T G C C G T A A T G C G C A T T C A G C T G A A C T G A C G T C A G T A G T C C G T A C A G T C T A G C A T G | VDR(NR),DR3/GM10855-VDR+vitD-ChIP-Seq(GSE22484)/Homer | 1e-2 | -4.639e+00 | 0.0259 | 781.0 | 3.71% | 416.9 | 3.42% | motif file (matrix) | svg |
